# Development of an improved inhibitor of Lats kinases to promote regeneration of mammalian organs

**DOI:** 10.1073/pnas.2206113119

**Published:** 2022-07-08

**Authors:** Nathaniel R. Kastan, Sanyukta Oak, Rui Liang, Leigh Baxt, Robert W. Myers, John Ginn, Nigel Liverton, David J. Huggins, John Pichardo, Matthew Paul, Thomas S. Carroll, Aaron Nagiel, Ksenia Gnedeva, A. J. Hudspeth

**Affiliations:** ^a^Howard Hughes Medical Institute and Laboratory of Sensory Neuroscience, The Rockefeller University, New York, NY 10065;; ^b^Tri-Institutional Therapeutics Discovery Institute, New York, NY 10021;; ^c^Department of Physiology and Biophysics, Weill Cornell Medical College of Cornell University, New York, NY 10065;; ^d^Bioinformatics Resource Center, The Rockefeller University, New York, NY 10065;; ^e^The Vision Center, Department of Surgery, Children’s Hospital Los Angeles, Los Angeles, CA 90027;; ^f^The Saban Research Institute, Children’s Hospital Los Angeles, Los Angeles, CA 90027;; ^g^Roski Eye Institute, Department of Ophthalmology, Keck School of Medicine, University of Southern California, Los Angeles, CA 90033;; ^h^Eli and Edythe Broad CIRM Center for Regenerative Medicine and Stem Cell Research, University of Southern California, Los Angeles, CA 90033;; ^i^Tina and Rick Caruso Department of Otolaryngology-Head and Neck Surgery, University of Southern California, Los Angeles, CA 90033

**Keywords:** cardiomyocyte, mouse, regeneration, retinal organoid, TRULI

## Abstract

The Hippo signaling pathway acts as a brake on regeneration in many tissues. This cascade of kinases culminates in the phosphorylation of the transcriptional cofactors Yap and Taz, whose concentration in the nucleus consequently remains low. Various types of cellular signals can reduce phosphorylation, however, resulting in the accumulation of Yap and Taz in the nucleus and subsequently in mitosis. We earlier identified a small molecule, TRULI, that blocks the final kinases in the pathway, Lats1 and Lats2, and thus elicits proliferation of several cell types that are ordinarily postmitotic and aids regeneration in mammals. In the present study, we present the results of chemical modification of the original compound and demonstrate that a derivative, TDI-011536, is an effective blocker of Lats kinases in vitro at nanomolar concentrations. The compound fosters extensive proliferation in retinal organoids derived from human induced pluripotent stem cells. Intraperitoneal administration of the substance to mice suppresses Yap phosphorylation for several hours and induces transcriptional activation of Yap target genes in the heart, liver, and skin. Moreover, the compound initiates the proliferation of cardiomyocytes in adult mice following cardiac cryolesions. After further chemical refinement, related compounds might prove useful in protective and regenerative therapies.

## Significance

Many organs regenerate through the proliferation of cells that replace those that have succumbed to aging or injury. However, proliferation is largely absent in certain critical organs, including the heart, central nervous system, and sensory organs such as the inner ear and retina. The Hippo-Yap biochemical signaling pathway, a cascade of proteins that—when active—inhibits cell division, constitutes one impediment to proliferation. We earlier identified a small molecule that interrupts Hippo-Yap signaling and thus relieves this block for some non-proliferating mammalian cells. We have chemically modified the original substance to yield a more potent analog that is effective for several hours and initiates the proliferation of lesioned heart-muscle cells. Compounds of this family might prove useful in regenerative therapies.

The highly conserved Hippo pathway is a key participant in numerous physiological processes and pathologies, including development, homeostasis, regeneration, cancer, and neurodegeneration (1–4). Hippo signaling integrates information from the cellular environment, including biomechanical cues, cell density, cell polarity, metabolic challenges, and signals such as Notch and Wnt. The core pathway comprises two pairs of core kinases. When activated by upstream signals, Mst1 and Mst2 phosphorylate Lats1 and Lats2; the latter kinases then phosphorylate the transcriptional coactivators Yap and Taz. Although those proteins can potentially initiate cell division by interacting in the nucleus with transcription factors of the Tead family, their activity depends on a dynamic flux between the cytoplasm and nucleus. Yap and Taz constantly shuttle between the two compartments (5), and their rates of movement depend both on their degree of phosphorylation and on factors such as mechanical force on the nucleus and modulation of the efflux pathway (6–8). When the rate of nuclear efflux falls well below that of influx, for example because the kinase cascade is inactive, the enhanced concentration of unphosphorylated Yap and Taz in the nucleus favors cell division (9–11).

Although transgenic approaches have elucidated much about the Hippo pathway, small molecules targeting the pathway offer a simplified avenue of investigation and could potentially have therapeutic utility. Over the last few years, a few compounds that target the Hippo pathway have been characterized. XMU-MP1, an inhibitor of the Mst kinases, augments the regeneration of organs in which the process occurs naturally, such as the liver and intestine (12). Quinolinols, which promote Yap-dependent transcription by stabilizing the interaction between Yap and Teads, enhance the healing of skin wounds in mice (13). An additional Lats inhibitor, GA-017, has recently been shown to augment the creation of mouse intestinal organoids in vitro (14).

In the hope of identifying substances that promote proliferation in supporting cells of the internal ear, we earlier screened small molecules for compounds that facilitate the nuclear enrichment of Yap. From this effort emerged an inhibitor of Lats kinases, TRULI, which proved effective not only for its original objective, but also for promoting the proliferation of inner-ear sensory epithelia, cultured primary cardiomyocytes, and human retinal organoids (15). We have subsequently conducted a medicinal-chemistry campaign to enhance the potency and drug-like properties of Lats inhibitors of this family and to improve their efficacy in vivo. Here we characterize TDI-011536, a derivative that demonstrates a marked improvement in potency and physicochemical properties and offers a simple paradigm for systemic Lats inhibition in several mammalian tissues in vitro and in vivo.

## Results

### Enhancement of TRULI's Activity through Medicinal Chemistry.

**Fig. 1.**
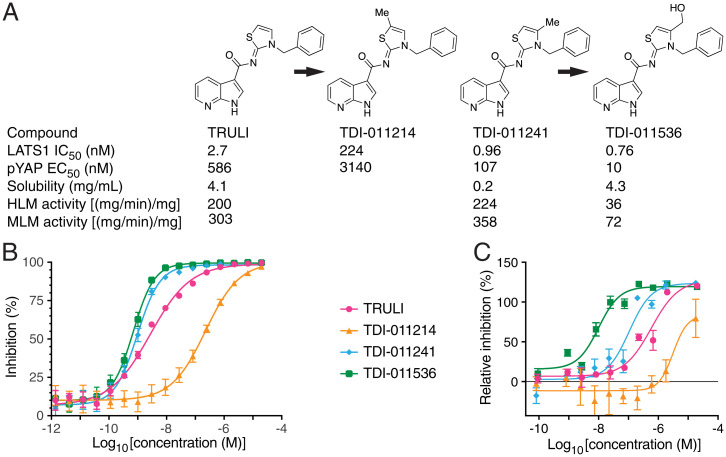
Development of an effective Lats kinase inhibitor. (*A*) The enhancement of the original Lats kinase inhibitor involved derivatization of the thiazolidine ring with a methyl and then a hydroxymethyl group. IC_50_ determinations were conducted in the presence of 2 mM ATP; aqueous solubility was measured at pH 6.8. HLM, human liver microsome; MLM, mouse liver microsome. (*B*) By an in vitro assay of Lats inhibition, methylation of the thiazolidine ring adjacent to the sulfur atom (TDI-011214) greatly diminished potency with respect to the original inhibitor TRULI. Methylation adjacent to the ring's nitrogen atom (TDI-011241) enhanced potency, and the hydroxymethyl derivative TDI-011536 was more effective still. (Error bars: SEMs [*n* = 4 measurements apiece in *n* = 4 independent experiments].) (*C*) The relative inhibition of Lats kinases was determined from the ratio of pYap to tYap in homogenates from treated HEK293A cells. Although the order of efficacy of the four compounds was identical, the performance of TDI-011536 was strikingly superior. (Error bars: SEM [*n* = 2 measurements apiece in *n* = 2 independent experiments].) For a total of 30 measurements for TDI-011536, EC_50_ = 40.8 ± 4.5 nM (mean ± SEM).

The original lead inhibitor of Lats kinases, TRULI, comprises a thiazolidine-2-imine core to which a hydrophobic benzyl moiety is linked through the ring's nitrogen atom. In addition, a 7-azaindole-3-carboxylate substituent is attached to the core at the imine nitrogen (Fig. 1*A*). Although TRULI demonstrated good biochemical potency, its cellular potency was modest and it exhibited poor stability on exposure to mouse liver microsomes. With the aim of identifying compounds of greater potency and better drug-like properties, we systematically modified TRULI. To assess the potency of the newly synthesized analogs, we measured their activity by an in vitro kinase assay and a cellular assay of Yap phosphorylation (15). The ATP concentration was increased from 10 μM to 2 mM because the increased potency of the compounds, which were predicted to act as ATP-competitive inhibitors of Lats kinases, prevented their evaluation in the original assay format. In addition, a value of 2 mM approximates the physiologically relevant ATP concentration.

Computational docking studies suggested that the azaindole moiety, like that of ATP, binds to the hinge region of Lats kinases (15). Initial exploration of structure-activity relationships revealed limited opportunities to modify this region. We therefore retained the azaindole hinge-binding element and focused on the thiazolidine core. Examination of the docking model of TRULI in Lats1 suggested that there was space to extend deeper into the pocket located beneath the thiazolidine-2*-*imine core. Exploration of this hypothesis with simple methyl substitution provided the 5- and 4-methyl substituted compounds TDI-011214 and TDI-011241, respectively (Fig. 1*A*). The 5-methyl substitution in the former compound potentially oriented the lipophilic methyl group toward a polar region. In line with this possibility, TDI-11214 suffered a significant loss of enzymatic potency. However, methylation in the adjacent 4 position yielded improvements in both biochemical and cellular potency.

Despite the improved activity of TDI-011241, the increased lipophilicity resulted in poor aqueous solubility at pH 6.8; moreover, the compound also suffered from low metabolic stability (Fig. 1*A*). We sought to improve those physical properties by incorporating polar substituents. Enhancing the 4-methyl with a hydroxyl group yielded TDI-011536, which gratifyingly offered a significant gain in cellular potency as well as improved aqueous solubility and metabolic stability. Examination of TDI-011536 in our docking model suggests that the pendant hydroxyl group makes a hydrogen bond with Asp789. Given the Lats concentration of 555 pM in the biochemical assay and the potential for both tight and slow binding inhibition, the affinities of compounds TDI-011241 and TDI-011536 are likely at or below the lower limit of accurate quantification, which implies true inhibitory activities significanly lower than 1 nM. This observation would explain the significan improvement in cellular potency relative to that of TRULI.

### Effect of TDI-011536 on Human Retinal Organoids.

**Fig. 2.**
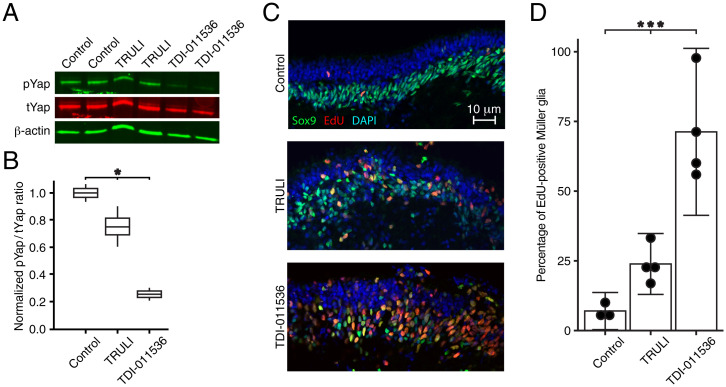
Proliferation of Müller glia in human retinal organoids after treatment with Lats kinase inhibitors. (*A*) An immunoblot for pYap and tYap after a 24 h incubation of human retinal organoids shows modest suppression of Yap phosphorylation after treatment with 10 μM TRULI and a profound effect of 3 μM TDI-011536 by comparison to controls containing DMSO, the solvation vehicle for the compounds. Each sample includes five organoids; β-actin serves as a loading control. (*B*) Quantification of the immunoblot by the ratio of pYap to tYap, both normalized to the DMSO control, confirms a significant effect of treatment with both compounds by one-way ANOVA; *P* = 0.019 for two experiments in each condition. (*C*) After 5 d of treatment, confocal immunofluorescence microscopy of sections from human retinal organoids reveals a substantial increase in cells doubly positive for EdU and Sox9, which represent proliferating Müller glia. (*D*) Quantification of the foregoing result demonstrates the significance of the effect by one-way ANOVA; *P* = 0.0003 for four experiments in each condition.

We have previously demonstrated that TRULI can decrease the phosphorylation of Yap and cause the proliferation of Müller glia in human retinal organoids in vitro (15). To test the potency of TDI-011536 in the same system, we applied the two compounds to retinal organoids derived from human induced pluripotent stem cells. After 24 h of Lats kinase inhibition with 10 μM TRULI, there was a significant 30% reduction in Yap phosphorylation as determined by the ratio of phospho-Yap (pYap) to total Yap (tYap) (Fig. 2*A* and *B* and *SI Appendix*, Fig. S1*A*). After treatment with 3 μM TDI-011536, Yap phosphorylation was reduced by almost 80% from the level in controls containing an identical amount of dimethyl sulfoxide (DMSO), the vehicle used to solubilize the compound. We next tested the effect of increased Yap signaling on proliferation in Müller glia, which we assessed in organoids cultured in the presence of the thymidine analog 5-ethynyl-2′-deoxyuridine (EdU). After a 5-d treatment with 10 μM TRULI, there was a robust, almost fivefold increase in the number of Sox9-positive Müller glia that incorporated EdU (Fig. 2*C* and *D*). By contrast, TDI-011536 treatment increased the number of doubly positive cells tenfold. In both instances, labeling for activated caspase 3 revealed little apoptosis (*SI Appendix*, Fig. S1*B*).

These data confirm that inhibition of Lats kinases stimulates Yap signaling and the proliferation of Müller glia in human retinal organoids and demonstrate that TDI-011536 is significantly more potent than TRULI in this system. In conjunction with the improved solubility and metabolic stability of the compound, the results advanced TDI-11536 as an appropriate tool compound for investigating LATS inhibition in vivo.

### In Vivo Activity of TDI-011536.

**Fig. 3.**
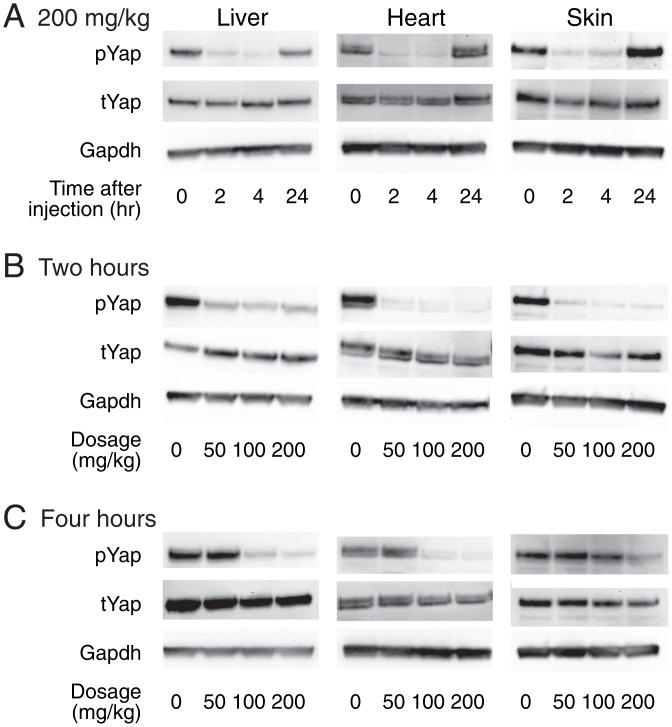
Engagement of target organs in vivo. (*A*) Immunoblots portray the amounts of Yap phosphorylated at residue S127 (pYap) and the total amounts of Yap (tYap) in the liver, heart, and skin. Injection of TDI-011536 at a dosage of 200 mg/kg greatly reduces the amount of pYap for at least 4 h after injection in all three organs. The levels return to control values within a day. Glyceraldehyde 3-phosphate dehydrogenase (Gapdh) is included in each instance as a loading control. (*B*) Two hours after injection, all three dosages of TDI-011536 largely suppress the amount of pYap. (*C*) By 4 h after injection, pYap has recovered following injections at 50 mg/kg. The concentration of pYap remains low in the heart and liver following injections at 100 mg/kg and 200 mg/kg, but largely recovers in the skin at the former dosage.

To explore the efficacy of TDI-011536 in living animals, we conducted intraperitoneal injections of mice with a relatively high dose of 200 mg/kg of body weight. The compound was dissolved in Kolliphor HS 15, a vehicle that was also injected into control animals. Immunoblot analysis of the heart, liver, and skin immediately after injection demonstrated the high concentration of phosphorylated Yap (pYap) characteristic of control conditions. Two or four hours after injection, however, all three organs in treated animals demonstrated a remarkable reduction in pYap relative to total Yap (tYap; Fig. 3*A*). This process was reversible; by eight hours, pYap levels returned to their baseline levels (*SI Appendix*, Fig. S2).

We next evaluated the effect of dosage de-escalation on the pharmacokinetics. We compared the original 200 mg/kg dose to injections of 100 mg/kg and 50 mg/kg. All three treatments evoked profound reductions of pYap in the three organs within 2 h of injection (Fig. 3*B*). After 4 h, however, the 50 mg/kg dose no longer diminished pYap significantly in any organ (Fig. 3*C*). The 100 mg/kg dose maintained the dephosphorylation of Yap through 4 h in the liver and heart, but failed to do so in the skin. These data together demonstrate the concentration dependence of TDI-011536 activity in vivo and suggest that an injection of 200 mg/kg provides over 4 h of Lats inhibition in the liver, heart, and skin.

### Effects of Lats Kinase Inhibition on Gene Expression.

**Fig. 4.**
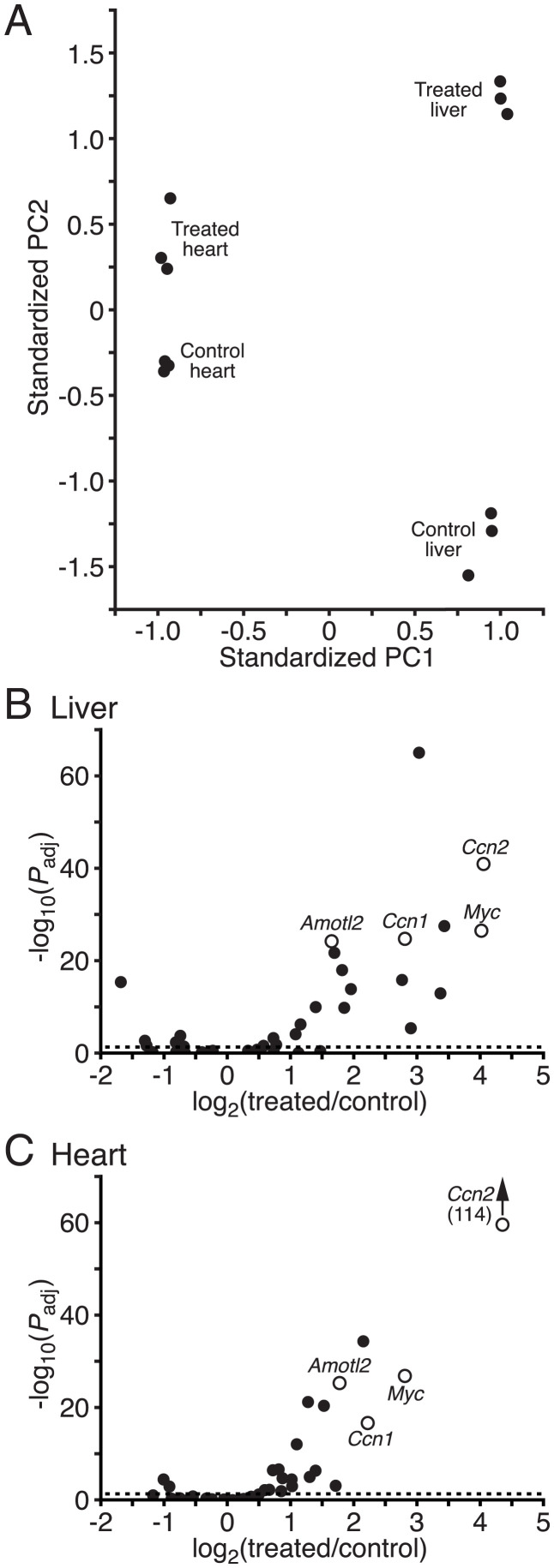
The effects of TDI-011536 treatment on gene expression. (*A*) Principal-components analysis of gene expression in the liver and heart indicates that the data are separated first on the basis of the respective organs, then in response to treatment with TDI-011536. The first principal component (PC1) captures 87.5% of the explained variance and the second (PC2) accounts for 3.8%. (*B*) A volcano plot demonstrates up-regulation of Yap target genes after exposure to TDI-011536 in the liver. Examples of well-characterized targets of Yap are indicated as hollow circles: *angiomotin-like 2* (*Amotl2*), *cellular communication network factor 1* (*Ccn1*), *cellular communication network factor 2* (*Ccn2*), and *myc proto-oncogene* (*Myc*) The dashed horizontal line represents an adjusted probability level of *P*_adj_ = 0.05. (*C*) The corresponding volcano plot for the heart reveals a similarly robust enhancement of expression of Yap target genes. Data relating to gene expression in response to TDI-011536 treatment are available at GEO (https://www.ncbi.nlm.nih.gov/geo/query/acccgi?acc=GSE196322).

To confirm that treatment with TDI-011536 activates Yap in vivo, we collected livers and hearts for RNA sequencing 4 h after injection at 200 mg/kg. Principal-components analysis of gene expression revealed that the samples clustered first by organ and second by treatment (Fig. 4*A*). We used gene set-enrichment analysis to examine changes in targets of Yap activity (16, 17). In both the liver and heart, genes characteristically activated upon Yap expression displayed significant enrichment after exposure to TDI-011536 (Fig. 4*B* and *C* and Table 1 and *SI Appendix*, Table S1). Although some genes associated with the G1/S and G2/M phases of mitosis changed significantly in the liver, the set as a whole was not significantly enriched in either organ (*SI Appendix*, Fig. S3*A*). This result might be expected after only a few hours of Yap activity, especially in the postmitotic heart.

**Table 1.** Up-regulation of selected Yap-target genes by TDI-011536

Gene	Liver			Heart		
	Control	Treated	Significance	Control	Treated	Significance
*Amotl2*	8.58 ± 0.05	10.21 ± 0.07	5 × 10^−25^	9.77 ± 0.09	11.53 ± 0.13	5 × 10^−26^
*Ccn1*	6.77 ± 0.06	9.50 ± 0.27	2 × 10^−25^	9.28 ± 0.16	11.46 ± 0.29	2 × 10^−17^
*Ccn2*	6.59 ± 0.14	10.55 ± 0.33	1 × 10^−41^	9.66 ± 0.18	14.02 ± 0.14	9 × 10^−115^
*Myc*	7.23 ± 0.32	10.67 ± 0.23	3 × 10^−28^	5.42 ± 0.18	8.15 ± 0.10	1 × 10^−27^

Each measurement shown is an rLog statistic that represents a modified log_2_ transformation regularized by variance stabilization. This approach accounts for the relationship between variance and mean in RNA-seq data by shrinking the observed variance for genes with low counts. By preventing the data from genes with low counts from becoming spread apart after logarithmic transformations, the procedure allows meaningful signals to better emerge from the background noise. Uncertainties are shown as SEMs for three measurements in each instance. Significance values were determined by two-tailed Wald tests.

To examine potential toxicity in these organs, we used gene set-enrichment analysis to inquire whether the gene ontology terms associated with inflammation (GO:0006954) or apoptosis (GO:0006915) were enriched after treatment. Although in both organs we observed an enhancement of genes associated with inflammation, we could not demonstrate enrichment of any daughter gene ontology terms; the effect might therefore be nonspecific. Although some genes associated with apoptosis were up-regulated, the entire group did not display significant enrichment (*SI Appendix*, Fig. S3*B*). Further gene-ontology analysis of the genes enriched or reduced in both categories failed to identify a clear directionality of the effect. These results suggest that 4 h treatment in vivo with TDI-011536 did not elicit significant damage in the organs examined. To gain an additional sense of categorical cellular effects, we examined the gene-ontology terms most significantly enriched after treatment. The lists of top hits included numerous categories that are widely associated with Hippo-Yap signaling (*SI Appendix*, Table S2).

### In Vivo Proliferative Effect of TDI-011536 on Cardiomyocytes.

Cardiomyocytes ordinarily display little or no proliferation in mature mammals; instead, cardiac lesions in mice evoke scarring (18). To ascertain whether TDI-011536 restores a capacity for proliferation, we therefore examined the effect of systemic administration of the compound on damaged hearts in mice. After optimizing the surgical approach and establishing an appropriate dosage regime, we conducted eight experiments on male 8-week-old mice. In each instance, we used a metal probe cooled in liquid nitrogen to create a small cryolesion on the right ventricle (18). We then inquired whether the cardiomyocytes near the affected area demonstrated proliferation, as marked by the incorporation of EdU.

**Fig. 5.**
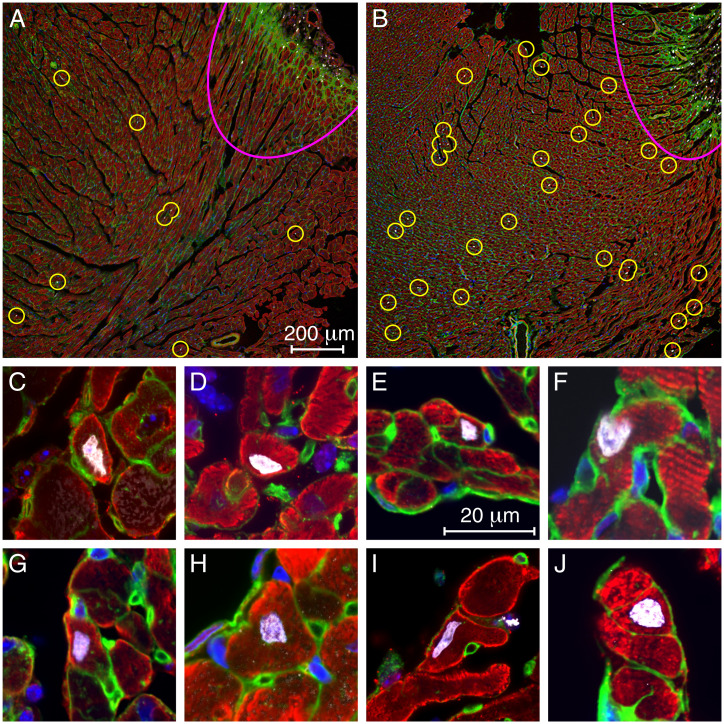
EdU incorporation into cardiomyocytes treated with TDI-011536. (*A*) At a distance from the lesion (pink arc at top right), a low-power micrograph of a representative section from a control animal shows only a few, small EdU-positive cells (circles). (*B*) After 3 d of treatment with TDI-011536, there are substantially more EdU-labeled cells outside the lesion, many of them large cardiomyocytes. (*C*–*J*) Individual EdU-labeled cardiomyocytes from treated animals are displayed. In all the images, cardiomyocytes are immunolabeled for troponin I or alpha-smooth muscle actin (red). Nuclei are stained with DAPI (blue) and those incorporating DNA precursors are additionally marked with EdU (white). By labeling membrane glycoproteins, wheat-germ agglutinin (green) helps to delineate the boundaries of cardiomyocytes. The small round profiles represent capillaries.

Two or three days' intraperitoneal dosing of six animals with 100 mg/kg of TDI-011536 resulted in the survival of all the mice for 2 wk with no apparent ill effects. Two control animals were lesioned and injected with vehicle on the identical schedule. Outside the actual lesions, where numerous cells proliferated in the course of scar formation, EdU-positive cells were rare in the controls and few if any labeled cardiomyocytes were observed (Fig. 5*A*). In treated animals, on the other hand, EdU-labeled cells were widely scattered throughout the myocardium (Fig. 5*B*). Examination of the tissue at high magnification revealed numerous labeled cardiomyocytes (Fig. 5*C*–*J*).

In 33 images from 13 sections from the hearts of the two animals treated with TDI-011536 for 3 d, the average density of EdU labeling was 17.0 ± 3.5 μm^−2^ (mean ± SD; *SI Appendix*, Table S3). By contrast, 28 images from 12 sections from controls yielded a labeling density of 5.6 ± 2.1 μm^−2^ (mean ± SD; *SI Appendix*, Table S4). Because the values for the two control samples were not statistically different, those data were combined. Both treated samples showed significantly more labeling than controls, one with *P* < 10^−9^ and the other with *P* < 10^−14^ by one-tailed *t* tests. Combining the experimental results as well yielded an overall significance of *P* < 10^−21^ (*SI Appendix*, Fig. S5).

## Discussion

Because of its ubiquitous role in suppressing cell division, the Hippo-Yap phosphorylation cascade represents a potential impediment to regenerative therapies. Although the pathway's activity might be modulated at several upstream levels, the key downstream targets are Lats1 and Lats2, paralogous protein kinases that directly modulate the activity of the transcriptional coactivators Yap and Taz. When unphosphorylated, Yap and Taz accumulate in the nucleus and enhance the expression of a variety of proteins associated with the cell cycle and mitosis. The small molecule TRULI, which blocks both Lats kinases, has therefore proven effective in vitro at triggering proliferation in mammalian organs as recalcitrant as the inner ear, retina, and heart (11).

In the present study, we have examined analogs of TRULI in an effort to determine which aspects of the molecule can be altered to enhance properties such as Lats inhibition, drug-like properties, and in vivo efficacy. A promising derivative, TDI-011536, demonstrates significant improvements in both potency and stability. Of equal importance, upon systemic administration the compound is not unduly toxic, reduces the phosphorylation of Yap for several hours in multiple organs in vivo, and evokes a modest degree of DNA synthesis in cardiomyocytes after focal cardiac damage.

Murine cardiomyocytes generally lose the capacity for proliferation by the first week after birth, but a burst of proliferation follows a week later owing to thyroid stimulation and some regenerative capacity persists for a few additional weeks (19–21). Although the majority of ventricular cardiomyocytes in mature mice are binucleated (22), the expression pattern of genes related to proliferation suggests that the minority population of mononucleated cells retains a greater proliferative capacity (23). Our results do not indicate the nuclear status of the cells that incorporate EdU and might therefore contribute to regeneration of damaged heart tissue. Moreover, we have not yet investigated the fate of the EdU-positive cells, which might undergo polyploidy or polynucleation without cytokinesis (19).

Although compounds related to TDI-011536 might have utility in regenerative therapies, accomplishing this would require overcoming several potential obstacles. In many instances, the suppression of Lats activity alone is unlikely to suffice for a proliferative effect (24). In experiments on the internal ear in vitro, for example, TRULI provokes robust mitosis in supporting cells of a vestibular organ, the utricle, yet fails to elicit such a response in the auditory organ of Corti (15). Even though compounds of this class might release one brake on cellular proliferation, there are doubtlessly others that would need to be independently inactivated.

Potential toxicity is a second concern. The human proteome contains over 500 protein kinases (25), many with similar binding sites for ATP. As a result, it is extraordinarily difficult to target a particular kinase without off-target effects. TRULI, the progenitor of TDI-011536, blocks several off-target kinases, but few of major importance (15). To realize any therapeutic potential of compounds of this class, it would be necessary to profile in detail their interaction with a wider variety of kinases and to conduct further medicinal chemistry in an effort to reduce side effects.

A related issue is the mechanism of dosage. Systemic administration of a compound such as TDI-011536 obviously risks deleterious effects in numerous organs, and indeed the present study was restricted in young mice by lethality related to intraperitoneal injection. However, in many instances it should be possible to limit this problem by focused administration of a compound, for example into the perilymph of the internal ear, the vitreous body of the eye, or the pericardium of the heart. Localized administration by a minipump or other apparatus for focal infusion might prove effective as well.

The duration of exposure is also an important consideration. Any treatment that suppresses the normal restrictions on cellular proliferation could prove dangerous as a result of unwanted hyperplasia or even carcinogenesis. An attractive feature of treatment with a substance related to TDI-011536, however, is that such compounds evoke proliferation within only a few days (15) and need not be administered for any longer period: in most instances, one or a few rounds of mitosis could restore a damaged organ's function. Moreover, although some hyperplasia was observed during treatment with a compound that blocks upstream components of the Hippo-Yap pathway, the Mst kinases, the affected organs reverted to normal size within days after exposure without an increased incidence of cancer during the subsequent year (12).

Two other characteristics of potential therapeutic agents could be improved by additional chemical intervention. It is likely that the efficacy of the compounds could be further enhanced: some inhibitors already in hand block Lats kinases at subnanomolar concentrations even in the presence of 2 mM ATP and suppress pYap with an EC_50_ in the low nanomolar range in HEK293A cells. Although greater potency might be associated with more off-target activity, there are likely compounds both more potent and more selective than TDI-011536. A second feature that could be improved is solubility. With few polar groups, TRULI and TDI-011536 are sparingly soluble in aqueous media. As further investigations—especially crystallography—clarify the interactions of such compounds with Lats kinases, it should become apparent at what sites polar groups might be added to enhance solubility and thus to facilitate the administration of potential drugs.

## Materials and Methods

Detailed methods are provided in *SI Appendix*, *SI Materials and Methods*.

In an effort to develop compounds that promote the proliferation of mammalian cells, we conducted a range of chemical modifications of a small molecule previously shown to block the activity of Lats kinases. As described in the earlier study (15), the resultant substances were assessed both by Lats inhibition in an enzymatic assay and by the suppression of Yap phosphorylation in HEK293A cells; we also examined cellular proliferation in retinal organoids derived from human induced pluripotent stem cells. To determine the effect of exposure in vivo to the most effective compound, TDI-011536, we conducted RNA sequencing on control and treated livers and hearts from Swiss Webster mice, then compared the resultant gene-expression profiles to known targets of Yap expression (16, 17). To assess the proliferation of cardiomyocytes, we injected the compound intraperitoneally into mice whose right ventricles had been cryolesioned with a metal probe cooled in liquid nitrogen (18).

## Supplementary Material

Supplementary File

## Data Availability

Gene-expression data have been deposited in Gene Expression Omnibus (GSE196322) (26). All study data are included in the article and/or *SI Appendix*.
