# Supplementary material for: Development of an improved inhibitor of Lats kinases to promote regeneration of mammalian organs
Source: Proc Natl Acad Sci U S A. 2022 Jul 8;119(28):e2206113119. doi: 10.1073/pnas.2206113119 (PMC9282237; doi:10.1073/pnas.2206113119)
Supplement: Supplementary File [file pnas.2206113119.sapp.pdf]

Supporting Information for

# Development of an improved inhibitor of Lats kinases to promote regeneration of mammalian organs

Nathaniel R. Kastan[a,†], Sanyukta Oak[a,†], Rui Liang[b], Leigh Baxt[b], Robert W. Myers[b], John Ginn[b], Nigel Liverton[b], David J. Huggins[b,c], John Pichardo[b], Matthew Paul[d], Thomas S. Carroll[d], Aaron Nagiel[e,f,g], Ksenia Gnedeva[h,i,‡], and A. J. Hudspeth[a,‡1]

[a] Howard Hughes Medical Institute and Laboratory of Sensory Neuroscience, The Rockefeller University, New York, NY 10065; [b] Tri-Institutional Therapeutics Discovery Institute, New York, NY 10021, USA; [c] Department of Physiology and Biophysics, Weill Cornell Medical College of Cornell University, New York, NY 10065, USA; [d] Bioinformatics Resource Center, The Rockefeller University, New York, NY 10065; [e] The Vision Center, Department of Surgery, Children's Hospital Los Angeles, Los Angeles, CA, USA; [f] The Saban Research Institute, Children's Hospital Los Angeles, Los Angeles, CA, USA; [g] Roski Eye Institute, Department of Ophthalmology, Keck School of Medicine, University of Southern California, Los Angeles, CA, USA; [h] Eli and Edythe Broad CIRM Center for Regenerative Medicine and Stem Cell Research, University of Southern California, Los Angeles, CA 90033, USA; [i] Tina and Rick Caruso Department of Otolaryngology—Head and Neck Surgery, University of Southern California, Los Angeles, CA 90033, USA

This file contains:

Materials and Methods
Figures S1 to S5
Tables S1 to S4
References

# SI Materials and Methods

## Assays of Lats kinase inhibition

The activity of compounds was tested against a functional component of Lats kinases in an enzymological assay (1). The substances' ability to reduce the phosphorylation of Yap was also tested in HEK 293 cells, again as described previously. For the latter assay, variations in the control responses sometimes led to negative values or values exceeding 100 % for the percentage of inhibition, but the dependence of the results on the concentrations of the test compounds was highly reproducible.

### Systemic administration of TDI-011536

procedures involved Swiss Webster mice (Charles River Laboratories) of both sexes and were conducted with the approval of Rockefeller University's Institutional Animal Care and Use Committee. Within each experiment, animals were age- and sex-matched and were randomly assigned to treated and control groups.

At a concentration of 5 mg/mL, TDI-011536 was emulsified by sonication into 10 % Kolliphor HS 15 (42966, Sigma) in phosphate-buffered saline solution (10010-023, Gibco). On each day of treatment, an animal received a single intraperitoneal injection of the mixture to yield a TDI-011536 dosage of 50-200 mg/kg. In the principal experiment with a dosage of 100 mg/kg, or 20 mL/kg, a typical animal of mass 30 g received an injection of 0.6 mL of solution.

When appropriate, 5-ethynyl-2′-deoxyuridine (EdU; E10187, Thermo Fisher) was administered intraperitoneally at a dosage of 5 mg/kg on each day of treatment with TDI-011536.

### Measurement of *in vivo* Lats inhibition

Fifty milligrams of heart, liver, or skin tissue was lysed on ice in radioimmunoprecipitation-assay (RIPA) buffer solution (BP-115, Boston BioProducts) with protease inhibitors (Halt Protease

Inhibitor Cocktail 87786, Thermo Fisher). After remaining on ice for 30 min, the samples were sonicated at low power for 2 min with 10 s exposures separated by 10 s interruptions, then centrifuged at 21,130 X g for 10 min at 4 °C. The supernatants were filtered and the lysates were immediately subjected to electrophoresis or stored at -80 °C.

A standard immunoblotting protocol was used. After lysates had been prepared, 10 mg of protein from the heart or liver or 20 mg of protein from the skin was resolved on a 4 %-12 % gradient bis-tris gel (NP0322, Thermo Fisher). The proteins were transferred to a PVDF membrane (1704156, BioRad) and blocked for one hour at room temperature (MB-070, Rockland). We used primary antibodies directed against Yap (101199, Santa Cruz Biotechnology), phosphorylated Yap S127 (4911, Cell Signaling Technology) and Gapdh (ab8245, Abcam), which were reconstituted at a dilution of 1:1000 in blocking solution. After a membrane had been exposed to primary antibodies overnight at 4 °C, it was subjected to three 5 min washes in tris-buffered saline solution (28358, Thermo Fisher) with 0.05 % Tween-20. Secondary antibodies conjugated to horseradish peroxidase (W401B and W402B, Promega) were applied at a dilution of 1:10,000 in the same solution for one hour at room temperature before activity was detected (SuperSignal West Pico PLUS 34580, Thermo Fisher). Images were acquired with an iBright FL1000 system (Invitrogen).

**Culture of human pluripotent stem cells and retinal organoids**

After induced pluripotent stem cells of the WTC-11 line (Coriell Institute for Medical Research, Camden, NJ) had been grown by standard methods, human retinal organoids were produced (2)· The effects of Lats kinase inhibition were analyzed as described previously (1). For the preparation of protein lysates after a 24-hour treatment in two independent assays, five organoids 225-280 days of age were sampled per experimental group. In four independent proliferation assays, the organoids were incubated in long-term culture medium supplemented with 10 μM EdU and 10 μM TRULI or 3 μM TDI-011536. The extent of proliferation was assessed by quantifying the percentage cells doubly positive for EdU and Sox9 after five days in culture.

**RNA sequencing**

Sequence and transcript coordinates for the murine mm10 UCSC genome and gene models were retrieved from the Bioconductor Bsgenome.Mmusculus.UCSC.mm10 (version 1.4.0) and TxDb.Mmusculus.UCSC.mm10.knownGene (version 3.4.0) Bioconductor libraries, respectively. Transcript expression was calculated with Salmon quantification software (version 0.8.2) (3); gene expression levels as transcripts per million and counts were retrieved with Tximport (version 1.8.0) (4). Normalization and rlog transformation of raw read counts in genes were performed with DESeq2 (version 1.20.0) (4). *P*-values were corrected for multiple testing by the Benjamini-Hochberg algorithm. Intersample variability was assessed with hierarchical clustering, and heat maps of intersample distances were constructed in the Pheatmap R package (version 1.0.10). Gene set-enrichment analysis was conducted through clusterProfiler (version 3.18.1) (5). Other plotting was performed using ggplot2 (version 3.3.3).

**Cryolesioning of the murine right ventricle**

Cardiac lesions were produced by a variant of a published method (6). Twenty minutes before surgery, an adult Swiss Webster mouse (Charles River Laboratories) four or eight weeks of age and of either sex was given buprenorphine subcutaneously at a dosage of 100 μg/kg for analgesia. The animal was anesthetized with 100 mg/kg of ketamine and 5 mg/kg of xylazine administered intraperitoneally in sterile water. The animal's mid-ventrum was shaved, chemically depilitated, and sterilized with a povidine-iodine swab. The area of the skin incision was infiltrated intradermally with 1 mg/kg of bupivacaine. Subsequent procedures were conducted beneath a surgical microscope and under sterile conditions in a HEPA-filtered, positive-pressure hood.

With the animal supine on a thermostatted heating pad at 40 °C, an oblique, 15 mm-long incision was made from the xiphoid process parallel to the left lower costal margin and about 3 mm caudal to it. After the abdominal musculature and peritoneum had been transected with scissors in the same pattern, a miniature retractor was introduced to separate the two

sides of the incision. This approach exposed the upper margin of the liver and provided a view of the beating heart and the base of the left lung through the transparent diaphragm.

A cryolesion of the right ventricle was created with a round aluminum rod of mass 1.44 g secured to a plastic handle. A total of 76 mm in length, the rod was 3.2 mm in diameter over a distance of 59 mm at the base and 2.15 mm in diameter over the 17 mm at the tip. The rod was cooled for 20 s by complete immersion in liquid nitrogen, after which the slightly rounded, polished tip was immediately pressed against the diaphragm near the middle of the roughly rectangular area of exposure of the heart. A constant force, estimated as 0.8 N by simulation with an electronic balance, was exerted for 20 s, after which the probe was withdrawn. A successful lesion was marked by a 2 mm disk of frozen diaphragm—which rapidly thawed—and often by a dark patch on the subjacent heart.

It proved important to avoid two possible complications. First, it was necessary to insert the probe at a steep enough angle with respect to the horizontal to avoid touching and lesioning the liver. And second, it was essential not to exert excessive force: if the diaphragm wrapped around the end of the probe, it adhered so strongly that its withdrawal occasionally perforated the diaphragm and caused an immediately fatal pneumothorax. After preliminary experiments, however, all of the five to ten mice operated on a given day survived the procedure and became active in a warmed cage 10-30 min after surgery. To maintain analgesia, each animal was administered an additional 100 μg/kg of buprenorphine subcutaneously at 12-hour intervals for each of the days on which TDI-011536 was injected.

**Histological preparation and immunolabeling of cardiac muscle**

After an animal had been anesthetized and sacrificed by cervical dislocation, its heart was rapidly removed. The site of the lesion was apparent as a 2 mm purple spot in the middle portion of the right ventricle (6). To facilitate the access of fixative, the base and apex of the heart were amputated and the lesioned segment was immersed for 16 hours in 4 % formaldehyde in phosphate-buffered saline solution (28906, Thermo Fisher). The specimen

was then immersed overnight at 4 ˚C in 30 % sucrose solution, then infiltrated with cryoprotectant (OCT), frozen, and sectioned at a thickness of 5-8 μm.

Mounted on a glass slide, each section was blocked in a humidified chamber for one hour at room temperature with 3 % bovine serum albumin (AB 2336846, Jackson), 5 % normal donkey serum (AB-2337258, Jackson), and 0.5 % Triton X-100 (93443, Sigma) in tris-buffered saline solution. For the detection of EdU, the sections were then subjected to click chemistry (Click-iT EdU imaging kit C10340, Thermo Fisher) for 30 min at room temperature. After a short rinse with phosphate-buffered saline solution, the sections were labeled overnight at 4 ˚C with Alexa Fluor 488-labeled wheatgerm agglutinin (1:200; W11261, Thermo Fisher) in combination with a rabbit polyclonal primary antiserum, either anti-cardiac troponin I (1:50; ab47003, Abcam) or anti-alpha smooth muscle actin (1:50; ab5694, Abcam). After slides has been washed three times with phosphate-buffered saline solution, Alexa-Fluor 555-labeled secondary antiserum (1:500; A32794, Invitrogen) diluted in blocking solution was added for one hour at room temperature followed by two washes with phosphate-buffered saline solution. Nuclei were stained with DAPI and sections were mounted with glass coverslips in a mounting medium (Prolong Gold Anti-fade P36934, Thermo Fisher).

Low-power images were obtained with a confocal microscope (LSM 780, Zeiss) equipped with a 10X plan-apochromatic objective lens of numerical aperture 0.45. The images were processed with Fiji (7) to estimate the density of EdU-labeled cells (SI Appendix, Fig. S4). High-power images were acquired on an inverted microscope (IX81, Olympus) equipped with a super-resolution fluorescence illumination system (VT-iSIM, VisiTech International) and a 60X silicone-oil-immersion objective lens of numerical aperture 1.3 (UPLSAPO60XS2, Olympus).

## SI Figures and Legends

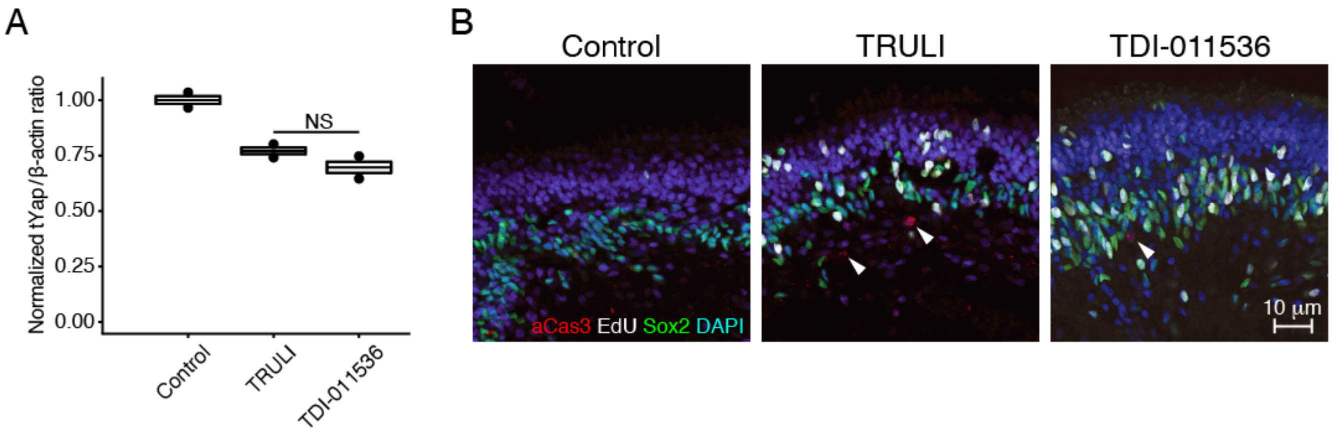


**Figure S1.** Control experiments relating to gene expression in retinal organoids. (*A*) Although in Figure 2*A* the tYap signals after TDI-011536 treatment appear weaker than those after exposure to TRULI, the effect stems from slightly lower loading of the gel. NS, not significant at $P > 0.05$. The systematically lower ratios in treated *versus* control samples might result from increased β-actin concentrations in mitotically active cells. (*B*) Although TDI-011536 causes extensive proliferation of Müller cells marked with Sox2 (green), as signaled by labeling with EdU (white), it elicits little apoptosis evidenced by activated caspase-3 (aCas3, red; arrowheads). DAPI (blue) marks all the nuclei.

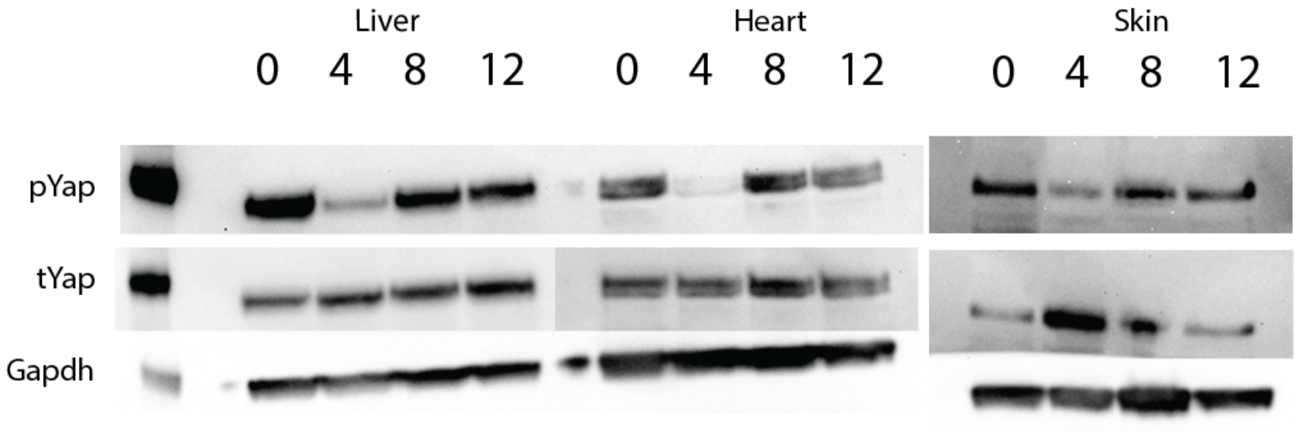


**Figure S2.** Recovery of pYap levels. Immunoblots portray the amounts of Yap phosphorylated at residue S127 (pYap) and the total amounts of Yap (tYap) in the liver, heart, and skin. Although intraperitoneal administration of TDI-011536 at a dosage of 200 mg/kg of body weight reduced the level of phosphorylation by four hours after injection, recovery was essentially complete by eight hours in all three organs. Glyceraldehyde 3-phosphate dehydrogenase (Gapdh) is included in each instance as a loading control.

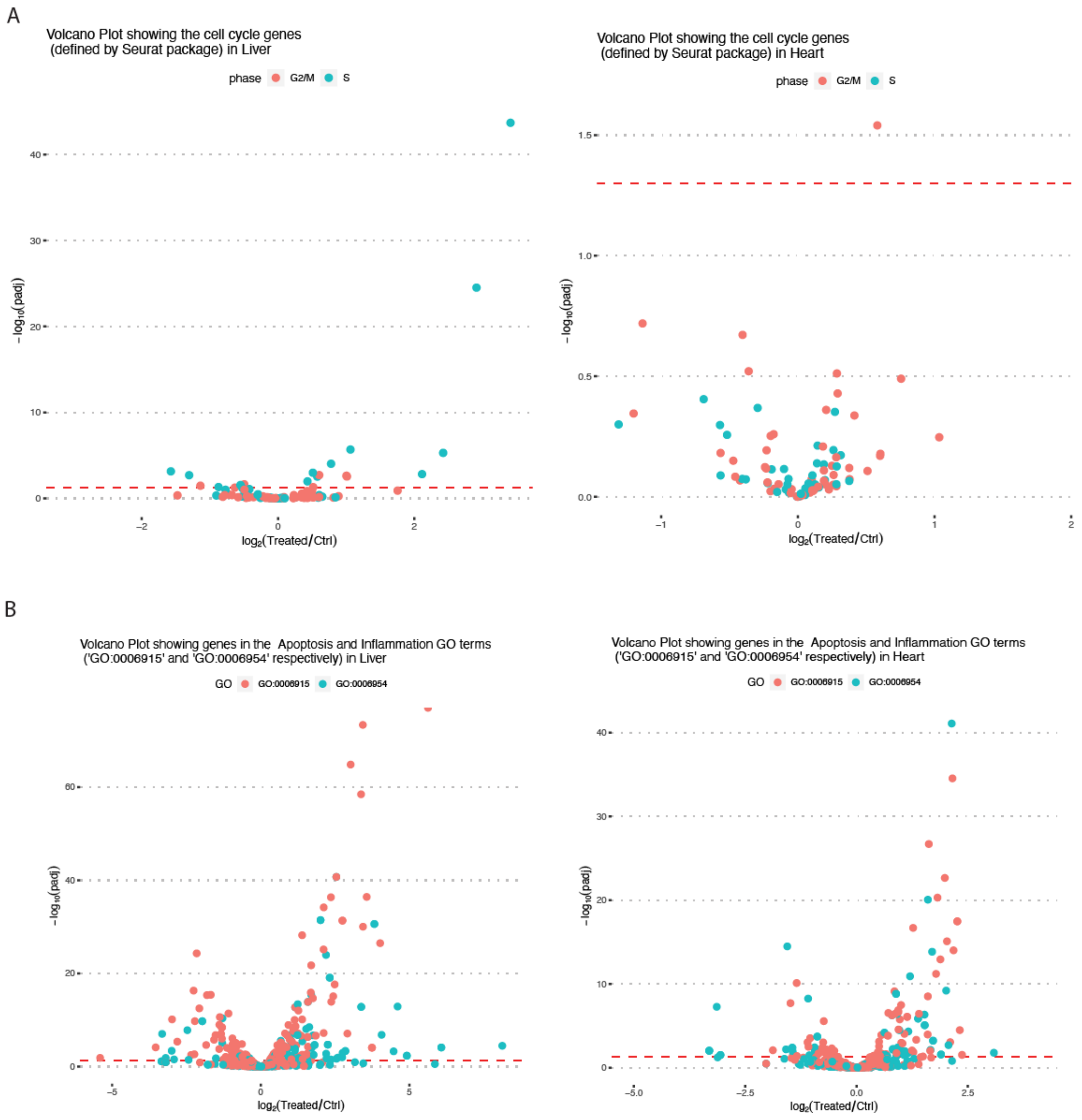


**Figure S3.** Effect of TDI-011536 on the expression in the liver and heart of genes associated with mitosis, inflammation, and apoptosis. These volcano plots demonstrate the individual genes from the respective GO term sets, with their fold changes presented as $\log_2$(treated/control) on the abscissa and the significance plotted on the ordinate as -$\log_{10}$(adjusted *P*-value). (*A*) Four hours after intraperitoneal administration of TDI-011536 at a dosage of 200 mg/kg, a small number of genes from the G2/M (red) and S (teal) GO terms are significantly enriched in the liver and heart, but the group as a whole is not significantly up-regulated by gene set-enrichment analysis. (*B*) After the same treatment, the Inflammation GO

set (teal) is significantly up-regulated in both liver and heart. Some of the apoptosis-associated genes (red) are significantly up-regulated, but gene set-enrichment analysis does not find the group as a whole enriched. Further gene-ontology analysis of the genes enriched or reduced in both categories fails to identify a clear directionality of the effects. Dotted red lines indicate adjusted *P*-values of 0.05.

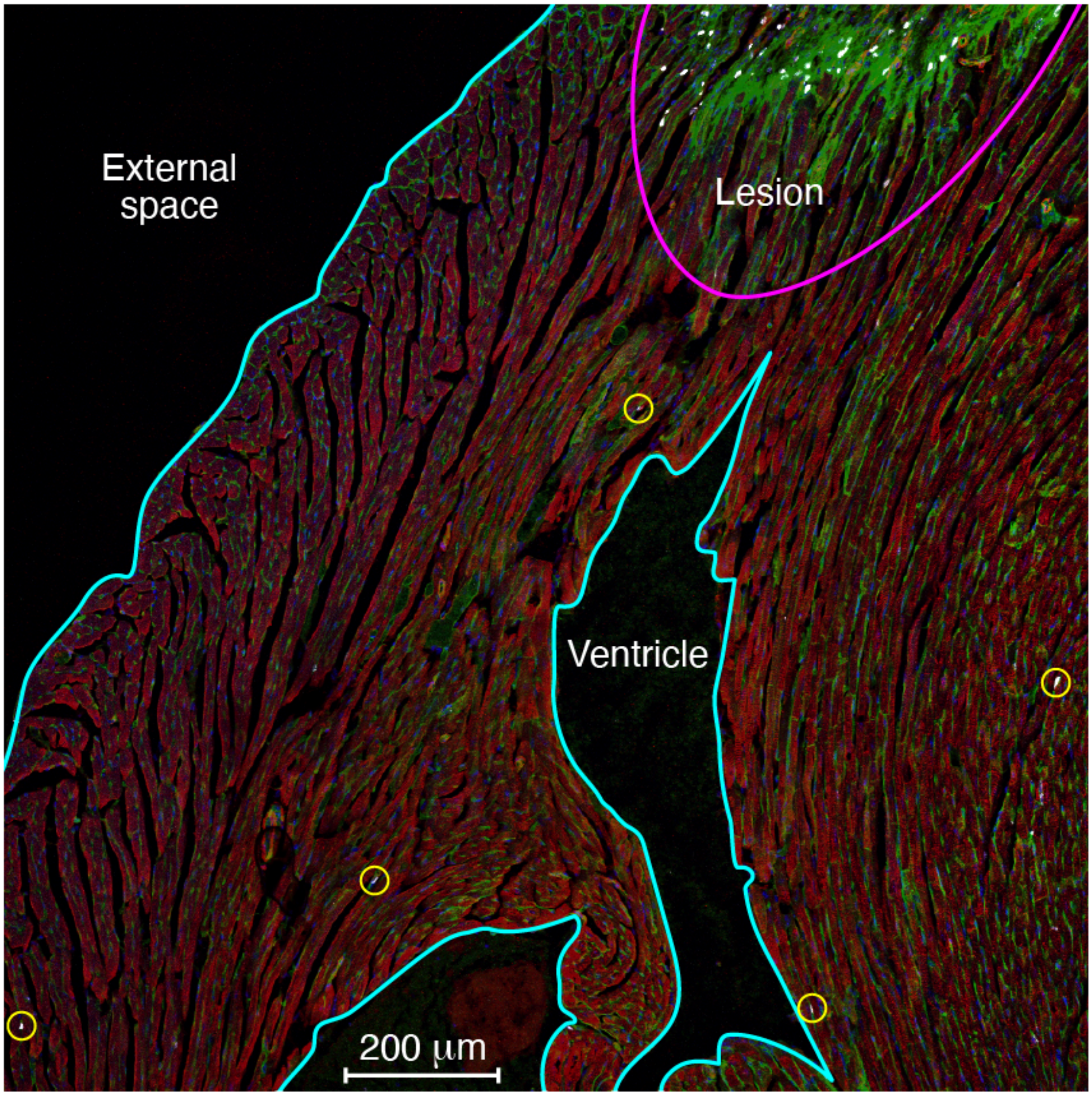


**Figure S4.** Procedure for estimation of Edu labeling density. In a low-power micrograph of right ventricular tissue from a control animal, a cryolesion (pink arc) is marked by enhanced labeling with wheat-germ agglutinin (green) owing to the elimination of the cells formerly in that area. Other areas free of muscle are outlined (light blue). The combined area of the lesion, the ventricular cavities, and the external space is 0.631 $mm^2$, or 31.4 % of the total image area of 2.008 $mm^2$. The 68.6 % of the image containing cardiac muscle and lying outside the lesion—an area of 1.377 $mm^2$—contains five EdU-positive cells (yellow circles); the labeling density is accordingly 3.63 $mm^{-2}$.

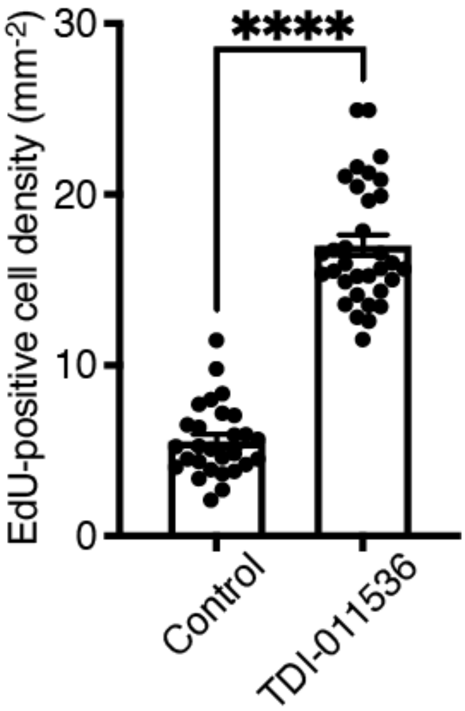


**Figure S5.** Graphic display of the data from Supporting Information Tables S3 and S4 shows the increase in the number of EdU-positive cells from 28 control and 33 TDI-011536-treated section of hearts. The combined data demonstrate enhanced labeling of treated specimens at the level $P < 10^{-21}$ by a single-tailed *t*-test with unequal variances.

## SI Tables

Table S1. Significantly upregulated Yap target genes

| Liver | Heart |
|---|---|
| *Sgk1* | ***Ccn2*** |
| ***Myc*** | *Sgk1* |
| ***Ccn2*** | ***Myc*** |
| *Gadd45b* | ***Amotl2*** |
| ***Ccn1*** | *Crim1* |
| ***Amotl2*** | *Emp2* |
| *Tns1* | ***Ccn1*** |
| *Tgm2* | *Ndrg1* |
| *Dusp1* | *Ankrd1* |
| *Dlc1* | *Dlc1* |
| *Ndrg1* | *Gadd45b* |
| *Thbs1* | *Asap1* |
| *Dab2* | *Marcks* |
| *Pmp22* | *Sh2d4a* |
| *Cavin2* | *Etv5* |
| *Tgfb2* | *Pmp22* |
| *Crim1* | *Thbs1* |
| *Itgb5* | *Tgfb2* |
| *Asap1* | *Fscn1* |
| *Etv5* | *Lhfp* |
| *Tk1* | *Dusp1* |
| *Gls* | *Col4a3* |
| *Sh2d4a* | *Cavin2* |
| *Agfg2* | *Ddah1* |

The table lists Yap target genes (8)(9) significantly upregulated at the level $P_{adj} < 0.05$ in the liver and heart four hours after TDI-011536 treatment. Bolded genes are highlighted in Table 1, and data relating to gene expression in response to TDI-011536 treatment are available at GEO (https://www.ncbi.nlm.nih.gov/geo/query/acc.cgi?acc=GSE196322).

Table S2. Gene-ontology terms displaying enrichment upon treatment

(*A*) Terms enriched in the liver

| Term identity | Description | Normalized enrichment score | Adjusted *P*-value |
|---|---|---|---|
| GO:0000165 | MAPK cascade | 1.72 | 2.69E-08 |
| GO:0001525 | angiogenesis | 1.83 | 2.69E-08 |
| GO:0001568 | blood vessel development | 1.75 | 2.69E-08 |
| GO:0001816 | cytokine production | 1.71 | 2.69E-08 |
| GO:0001944 | vasculature development | 1.74 | 2.69E-08 |
| GO:0006364 | rRNA processing | 2.26 | 2.69E-08 |
| GO:0006396 | RNA processing | 1.87 | 2.69E-08 |
| GO:0007155 | cell adhesion | 1.63 | 2.69E-08 |
| GO:0016072 | rRNA metabolic process | 2.22 | 2.69E-08 |
| GO:0016477 | cell migration | 1.64 | 2.69E-08 |
| GO:0022610 | biological adhesion | 1.61 | 2.69E-08 |
| GO:0022613 | ribonucleoprotein complex biogenesis | 2.31 | 2.69E-08 |
| GO:0023014 | signal transduction by protein phosphorylation | 1.73 | 2.69E-08 |
| GO:0030334 | regulation of cell migration | 1.67 | 2.69E-08 |
| GO:0032270 | positive regulation of cellular protein metabolic process | 1.54 | 2.69E-08 |
| GO:0040012 | regulation of locomotion | 1.65 | 2.69E-08 |
| GO:0042254 | ribosome biogenesis | 2.36 | 2.69E-08 |
| GO:0042981 | regulation of apoptotic process | 1.61 | 2.69E-08 |
| GO:0043067 | regulation of programmed cell death | 1.61 | 2.69E-08 |
| GO:0048514 | blood vessel morphogenesis | 1.81 | 2.69E-08 |
| GO:0060548 | negative regulation of cell death | 1.67 | 2.69E-08 |
| GO:0072358 | cardiovascular system development | 1.73 | 2.69E-08 |
| GO:1901700 | response to oxygen-containing compound | 1.54 | 2.69E-08 |
| GO:2000145 | regulation of cell motility | 1.67 | 2.69E-08 |
| GO:0043069 | negative regulation of programmed cell death | 1.64 | 3.26E-08 |

(*B*) Terms enriched in the heart

| Term identity | Description | Normalized enrichment score | Adjusted *P*-value |
|---|---|---|---|
| GO:0007155 | cell adhesion | 1.55 | 8.07E-08 |
| GO:0009719 | response to endogenous stimulus | 1.54 | 8.07E-08 |
| GO:0016477 | cell migration | 1.58 | 8.07E-08 |
| GO:0022610 | biological adhesion | 1.54 | 8.07E-08 |
| GO:0030334 | regulation of cell migration | 1.63 | 8.07E-08 |
| GO:0040012 | regulation of locomotion | 1.61 | 8.07E-08 |
| GO:0051270 | regulation of cellular component movement | 1.59 | 8.07E-08 |
| GO:2000145 | regulation of cell motility | 1.61 | 8.07E-08 |
| GO:0035295 | tube development | 1.55 | 1.45E-07 |
| GO:0031589 | cell-substrate adhesion | 1.90 | 2.36E-07 |
| GO:0042981 | regulation of apoptotic process | 1.48 | 3.26E-07 |
| GO:0003013 | circulatory system process | 1.77 | 3.74E-07 |
| GO:0008015 | blood circulation | 1.78 | 3.74E-07 |
| GO:0007167 | enzyme linked receptor protein signaling pathway | 1.59 | 4.15E-07 |
| GO:0044057 | regulation of system process | 1.69 | 4.44E-07 |
| GO:0043067 | regulation of programmed cell death | 1.47 | 4.44E-07 |
| GO:0001932 | regulation of protein phosphorylation | 1.48 | 9.99E-07 |
| GO:1903522 | regulation of blood circulation | 1.95 | 1.54E-06 |
| GO:0042325 | regulation of phosphorylation | 1.45 | 1.66E-06 |
| GO:1901566 | organonitrogen compound biosynthetic process | 1.43 | 4.23E-06 |
| GO:0051272 | positive regulation of cellular component movement | 1.66 | 4.69E-06 |
| GO:0023014 | signal transduction by protein phosphorylation | 1.59 | 4.87E-06 |
| GO:0040017 | positive regulation of locomotion | 1.64 | 5.24E-06 |
| GO:0030335 | positive regulation of cell migration | 1.66 | 5.24E-06 |
| GO:0001944 | vasculature development | 1.57 | 5.42E-06 |

The lists portray the top twenty-five gene-ontology terms for the liver (*A*) and heart (*B*), ranked by significance, enriched in RNA-sequencing data four hours after intraperitoneal administration of TDI-011536 at 200 mg/kg.

Table S3. Density of EdU-labeled cells in TDI-011536-treated preparations.

| Animal label | Slide label | Image number | Nonmuscle area (mm²) | Muscle area (mm²) | Labeled cell count | Density (mm⁻²) |
|---|---|---|---|---|---|---|
| | | | | | | |
| Specimen 1 | AB | 1.1 | 0.239 | 1.769 | 24 | 13.57 |
| | | 1.2 | 0.216 | 1.792 | 30 | 16.74 |
| | | 1.4 | 0.810 | 1.198 | 25 | 20.87 |
| | | 3.1 | 0.620 | 1.388 | 23 | 16.57 |
| | AC | 1.1 | 0.127 | 1.881 | 28 | 14.89 |
| | AG | 1.2 | 0.341 | 1.667 | 21 | 12.60 |
| | | 2.2 | 1.049 | 0.959 | 15 | 15.64 |
| | AK | 1.1 | 0.443 | 1.565 | 18 | 11.50 |
| | M | 1.2 | 0.525 | 1.483 | 19 | 12.81 |
| | | 2.1 | 0.223 | 1.785 | 24 | 13.45 |
| | | 3.1 | 0.497 | 1.511 | 23 | 15.22 |
| | S | 3.1 | 0.990 | 1.018 | 22 | 21.61 |
| | AV | 1.1 | 0.544 | 1.464 | 21 | 14.34 |
| | | 3.1 | 0.677 | 1.331 | 18 | 13.52 |
| | | | | | | |
| Specimen 2 | AB | 2.1 | 0.809 | 1.199 | 18 | 15.01 |
| | | 2.2 | 0.104 | 1.904 | 34 | 17.86 |
| | AG | 1.1 | 0.435 | 1.573 | 26 | 16.53 |
| | | 1.2 | 0.519 | 1.489 | 21 | 14.10 |
| | | 2.1 | 0.409 | 1.599 | 27 | 16.89 |
| | K | 1.1 | 0.629 | 1.379 | 21 | 15.23 |
| | | 1.2 | 0.318 | 1.690 | 27 | 15.98 |
| | | 2.1 | 0.444 | 1.564 | 32 | 20.46 |
| | | 2.2 | 0.301 | 1.707 | 34 | 19.92 |
| | | 3.1 | 0.250 | 1.758 | 37 | 21.05 |
| | | 3.2 | 0.204 | 1.804 | 28 | 15.52 |
| | P | 1.1 | 0.365 | 1.643 | 41 | 24.95 |
| | | 1.2 | 0.227 | 1.781 | 35 | 19.65 |
| | S | 1.1 | 0.443 | 1.565 | 24 | 15.34 |
| | | 1.2 | 0.730 | 1.278 | 20 | 15.65 |
| | | 2.1 | 0.163 | 1.845 | 41 | 22.22 |
| | | 2.2 | 0.220 | 1.788 | 38 | 21.25 |
| | | 3.1 | 0.190 | 1.818 | 29 | 15.95 |
| | | 3.2 | 0.365 | 1.643 | 41 | 24.95 |

Each image captured a field of 1417 $\mu$m X 1417 $\mu$m and therefore a total area of 2.008 mm$^2$. The density of labeled cells in 14 images from Specimen 1 was 15.2 ± 3.0 mm$^{-2}$ and that in 19 images from Specimen 2 was 18.3 ± 3.4 mm$^{-2}$ (means ± SDs). The two results were

significantly different at the level $P = 0.0088$ by a double-tailed *t*-test with unequal variances. Combining the data yielded a density of 17.0 ± 3.5 $mm^{-2}$ (mean ± SD).

Table S4. Density of EdU-labeled cells in control preparations.

| **Animal label** | **Slide label** | **Image number** | **Nonmuscle area (mm²)** | **Muscle area (mm²)** | **Labeled cell count** | **Density (mm⁻²)** |
|---|---|---|---|---|---|---|
| | | | | | | |
| Specimen 3 | AA | 1.1 | 0.582 | 1.426 | 11 | 7.71 |
| | | 1.2 | 0.237 | 1.771 | 8 | 4.52 |
| | | 2.1 | 0.338 | 1.670 | 12 | 7.19 |
| | | 3.1 | 0.448 | 1.560 | 13 | 8.33 |
| | AB | 1.1 | 0.631 | 1.377 | 5 | 3.63 |
| | | 2.1 | 1.018 | 0.990 | 7 | 7.07 |
| | AH | 1.1 | 0.332 | 1.676 | 7 | 4.18 |
| | | 2.1 | 0.864 | 1.144 | 5 | 4.37 |
| | AY | 1.2 | 1.015 | 0.993 | 4 | 4.03 |
| | P | 1.1 | 0.522 | 1.486 | 5 | 3.36 |
| | | | | | | |
| Specimen 4 | AA | 1.1 | 0.880 | 1.128 | 9 | 7.98 |
| | AE | 1.1 | 0.771 | 1.237 | 6 | 4.85 |
| | | 1.2 | 0.874 | 1.134 | 13 | 11.46 |
| | | 3.1 | 0.289 | 1.719 | 9 | 5.24 |
| | | 3.2 | 0.708 | 1.300 | 6 | 4.62 |
| | AG | 1.1 | 0.908 | 1.100 | 7 | 6.36 |
| | | 2.1 | 0.680 | 1.328 | 7 | 5.27 |
| | D | 1.1 | 0.952 | 1.056 | 4 | 3.79 |
| | | 1.2 | 0.933 | 1.075 | 7 | 6.51 |
| | | 2.1 | 0.995 | 1.013 | 6 | 5.92 |
| | T | 1.1 | 1.065 | 0.943 | 2 | 2.12 |
| | | 1.2 | 1.165 | 0.843 | 5 | 5.93 |
| | X | 1.1 | 0.475 | 1.533 | 15 | 9.78 |
| | | 2.1 | 1.235 | 0.773 | 3 | 3.88 |
| | | 3.1 | 0.451 | 1.557 | 7 | 4.50 |
| | | 3.2 | 0.772 | 1.236 | 7 | 5.66 |
| | Z | 1.1 | 0.831 | 1.177 | 6 | 5.10 |
| | | 2.1 | 0.897 | 1.111 | 3 | 2.70 |

Each image captured a field of 1417 μm x 1417 μm and therefore a total area of 2.008 $mm^2$. The density of labeled cells in ten images from Specimen 3 was 5.4 ± 1.9 $mm^{-2}$ and that in 18 images from Specimen 4 was 5.7 ± 2.3 $mm^{-2}$ (means ± SDs). The two results were not significantly different at the level $P = 0.80$ by a double-tailed *t*-test with unequal variances. Combining the data yielded a density of 5.6 ± 2.1 $mm^{-2}$ (mean ± SD).

## SI References


1. N. Kastan, *et al.*, Small-molecule inhibition of Lats kinases may promote Yap-dependent proliferation in postmitotic mammalian tissues. *Nat. Commun.* **12**, 3100 (2021).
2. X. Zhong, *et al.*, Generation of three-dimensional retinal tissue with functional photoreceptors from human iPSCs. *Nat. Commun.* **5**, 4047 (2014).
3. R. Patro, G. Duggal, M. I. Love, R. A. Irizarry, C. Kingsford, Salmon provides fast and bias-aware quantification of transcript expression. *Nat. Methods* **14**, 417–419 (2017).
4. M. I. Love, J. B. Hogenesch, R. A. Irizarry, Modeling of RNA-seq fragment sequence bias reduces systematic errors in transcript abundance estimation. *Nat. Biotechnol.* **34**, 1287–1291 (2016).
5. G. Yu, L.-G. Wang, Y. Han, Q.-Y. He, clusterProfiler: an R package for comparing biological themes among gene clusters. *Omics J. Integr. Biol.* **16**, 284–287 (2012).
6. P. Grisel, *et al.*, The MRL mouse repairs both cryogenic and ischemic myocardial infarcts with scar. *Cardiovasc. Pathol. Off. J. Soc. Cardiovasc. Pathol.* **17**, 14–22 (2008).
7. J. Schindelin, *et al.*, Fiji: an open-source platform for biological-image analysis. *Nat. Methods* **9**, 676–682 (2012).
8. M. Cordenonsi, *et al.*, The Hippo transducer TAZ confers cancer stem cell-related traits on breast cancer cells. *Cell* **147**, 759–772 (2011).
9. F. Zanconato, *et al.*, Genome-wide association between YAP/TAZ/TEAD and AP-1 at enhancers drives oncogenic growth. *Nat. Cell Biol.* **17**, 1218–1227 (2015).
